# Treatment resistance factors associated with Talaporfin sodium photodynamic therapy for local control after chemoradiotherapy for esophageal cancer

**DOI:** 10.1007/s10388-025-01177-w

**Published:** 2026-01-16

**Authors:** Masashi Tamaoki, Akira Yokoyama, Chikatoshi Katada, Kenshiro Hirohashi, Yosuke Mitani, Yusuke Amanuma, Takahiro Horimatsu, Hirokazu Higuchi, Natsuko Yamahigashi, Motoo Nomura, Hiroyuki Inoo, Katsuyuki Sakanaka, Mitsuhiro Nikaido, Takahiro Shimizu, Yo Kishimoto, Shigeru Tsunoda, Kazutaka Obama, Shinya Ohashi, Manabu Muto

**Affiliations:** 1 Department of Medical Oncology, Kyoto University Graduate School of Medicine, 54 Kawahara-Cho, Shogoin, Sakyo-Ku, Kyoto, 606-8507 Japan; 2 Department of Immuno-Oncology PDT, Kyoto University Graduate School of Medicine, Kyoto, Japan; 3 Department of Gastroenterology and Hepatology, Kitano Hospital, Osaka, Japan; 4 Department of Clinical Trial Promotion, Chiba Cancer Center, Chiba, Japan; 5 Department of Medical Equipment, Kyoto University Hospital, Kyoto, Japan; 6 Department of Radiation Oncology and Image-Applied Therapy, Kyoto University Graduate School of Medicine, Kyoto, Japan; 7 Department of Gastroenterology and Hepatology, Kyoto University Graduate School of Medicine, Kyoto, Japan; 8 Department of Otolaryngology-Head and Neck Surgery, Kyoto University Graduate School of Medicine, Kyoto, Japan; 9 Department of Surgery, Kyoto University Graduate School of Medicine, Kyoto, Japan

**Keywords:** Chemoradiotherapy, Esophageal cancer, Esophageal stenosis, Local neoplasm recurrence, Photodynamic therapy

## Abstract

**Background:**

Talaporfin sodium photodynamic therapy (Talaporfin-PDT) shows a high local complete response (L-CR) rate for local failure after chemoradiotherapy in esophageal cancer; however, some patients do not achieve L-CR. We investigated the treatment resistance factors associated with Talaporfin-PDT for local control.

**Methods:**

This study included 55 consecutive patients with ycT1-2 esophageal cancer who received Talaporfin-PDT. We investigated the L-CR rate, cumulative survival rate according to local effect, and treatment resistance factors associated with Talaporfin-PDT for local control using a multivariate logistic regression model.

**Results:**

The L-CR rate was 65.5% (95% confidence interval [CI]: 52.3–76.6). During the median follow-up of 17.8 months (range, 1.7–69.3), the 1-year overall survival (OS), progression-free survival (PFS), and disease-specific survival (DSS) rates were significantly higher in patients who achieved L-CR than in those who did not: 97.0% vs. 75.6% (*P* = 0.008), 64.6% vs. 0% (*P* < 0.001), and 97.0% vs. 85.7% (*P* = 0.005), respectively. In the multivariate logistic regression model, esophageal stenosis before Talaporfin-PDT was an independent predictor of local failure after Talaporfin-PDT (odds ratio: 5.626, 95% CI: 1.051–30.124, *P* = 0.044). The 1-year OS and DSS rates were significantly lower in patients with stenosis before Talaporfin-PDT than in those without: 75.0% vs. 92.4% (*P* = 0.015) and 85.7% vs. 94.6% (*P* = 0.015), respectively.

**Conclusions:**

Esophageal stenosis before Talaporfin-PDT was associated with resistance to local control and poor survival. The indications for the use of Talaporfin-PDT in patients with stenosis should be considered carefully.

**Supplementary Information:**

The online version contains supplementary material available at 10.1007/s10388-025-01177-w.

## Introduction

Esophageal cancer is the sixth most common cause of cancer death worldwide [1]. Although definitive chemoradiotherapy (CRT) is a curative treatment option for locally advanced esophageal cancer patient who do not wish to undergo surgery [2], local failure after CRT is high and has been major problem to achieve cure [3, 4]. Salvage surgery and endoscopic resection (ER) are indicated for local failure after CRT. However, salvage surgery could not preserve the organ and has a risk of severe complications including treatment-related death [5–7]. On the other hand, the salvage ER is a less-invasive curative treatment option which can preserve the organ; however. its indications are limited to treatment of mucosal lesions [8–10]. Chemotherapy such as taxane or immune checkpoint inhibitors may be selected as second-line chemotherapy for local failure after CRT. However, the complete response (CR) rate of such chemotherapy is quite low at less than 10% [11–13].

We previously reported that talaporfin sodium photodynamic therapy (Talaporfin-PDT) is an effective and safe treatment option as an organ-preservation strategy in patients with local failure after CRT for esophageal cancer [14]. The indications for Talaporfin-PDT are the presence of a residual or recurrent esophageal cancer localized shallower than the muscularis propria, no indications for salvage surgery because of an unfavorable general condition, and the patient’s refusal of salvage surgery. The local complete response (L-CR) rate for Talaporfin-PDT was reported as 69–88.5%, and life-threatening severe adverse events was not reported [14, 15]. Patients who achieve L-CR after PDT significantly improved survival compared with those who do not achieve L-CR [15, 16].

If local control can be achieved, the prognosis can be improved. Therefore, in this study, we investigated the treatment resistance factors for local control of Talaporfin-PDT in patients with esophageal cancer treated with CRT.

## Methods

### Participants

Patients who were initially treated with Talaporfin-PDT from October 2015 through April 2022 and who met the following criteria were retrospectively collected in our hospital. The inclusion criteria were as follows: (1) residual or recurrent esophageal cancer after CRT, (2) tumors pathologically diagnosed as squamous cell carcinoma or adenocarcinoma, (3) lesions limited to ycT1 or ycT2, and (4) no regional lymph node or distant metastases identified using computed tomography (CT). The exclusion criteria were as follows: (1) metachronous esophageal cancer, (2) newly diagnosed esophageal cancer in the radiation field used to treat other diseases, (3) metastasis in the esophagus, 4) history of treatment with porfimer sodium-PDT, (5) local failure after ER followed by prophylactic adjuvant CRT, or (6) no evaluation of local efficacy after treatment.

Residual esophageal cancer was defined as the presence of a non-CR lesion after CRT, recurrent esophageal cancer was defined as local recurrence after achieving CR for CRT, and residual or recurrent esophageal cancer was defined as local failure. Endoscopic findings before Talaporfin-PDT were classified into superficial type and submucosal tumor type and ulcerated type [17].

Written informed consent was obtained from all patients for the Talaporfin-PDT procedures.

### Study variables

The study variables were as follows: (1) L-CR rate after Talaporfin-PDT; (2) clinical characteristics (gender, age, ECOG performance status, cTNM stage, histology, initial treatment, tumor location, endoscopic findings, ycT stage, maximum tumor diameter on endoscopic images, circumference of tumor, esophageal stenosis, laser dose) according to local effect after Talaporfin-PDT; (3) cumulative survival rates (overall survival [OS], progression-free survival [PFS], and disease-specific survival [DSS]) according to local effect after Talaporfin-PDT; (4) adverse events associated with Talaporfin-PDT; (5) treatment resistance factors associated with Talaporfin-PDT for local control using a multivariate logistic regression model; and (6) cumulative survival rates (OS, PFS, and DSS) in patients with or without factors associated with resistance.

### Talaporfin-PDT

Talaporfin-PDT was applied by intravenous administration of 40 mg/m^2^ talaporfin sodium. The lesion was irradiated with a diode laser at a wavelength of 664 nm, 4 h after the administration of talaporfin sodium. The diode laser was delivered via a frontal light distributor through an endoscope. The fluence of the diode laser was set at 100 J/cm^2^, and the fluence rate was 150 mW/cm^2^. According to the size of the lesion, multiple treatment fields were overlapped to cover the whole lesion. Endoscopic observation was performed on the day after Talaporfin-PDT. If an insufficiently irradiated area was found, additional diode laser irradiation was performed as described previously [14]. Patients were hospitalized in a room maintained at less than 500 lx for 2 weeks and advised to avoid direct sun exposure for 4 weeks after the administration of talaporfin sodium.

### Evaluation of clinical stage

The clinical stage was determined according to the TNM classification of the International Union Against Cancer, 8th edition [18]. The ycT stage before Talaporfin-PDT was evaluated using endoscopic ultrasound (EUS). If evaluating the ycT stage by EUS was difficult, the invasive depth of the residual or recurrent esophageal cancer was evaluated by conventional endoscopy.

### Esophageal stenosis before Talaporfin-PDT

**Fig. 1:**
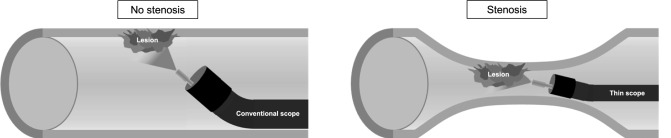
Talaporfin sodium photodynamic therapy (Talaporfin-PDT) in patients with esophageal stenosis. When using a thin endoscope to irradiate irradiating lesions within a stenotic area with the thin scope, it is technically difficult to maintain a vertical view of the lesion and an appropriate distance due to the narrow and often deformed lumen. These factors may contribute to the reduced treatment response observed in patients with pre-treatment stenosis. Therefore, irradiation must be performed at an inadequate oblique angle, and effective irradiation is not feasible

Esophageal stenosis was defined as the inability of a conventional scope (tip diameter < 10 mm) to pass from the primary site to the distal side. In this study, esophageal stenosis was analyzed as a situation in which all or part of an irradiated lesion was located within the stenosis. Figure 1 demonstrates representative image where the irradiation site overlaps with the area of stenosis. In patients with esophageal stenosis, when irradiation was difficult using a conventional scope, a thin scope was advanced into the esophageal stricture to irradiate the lesion. Esophageal stenosis before Talaporfin-PDT” that EG-L580NW (Fujifilm medical Co., Ltd., Tokyo, Japan) was used for PDT with the thin scope.

### Treatment evaluation and surveillance

After Talaporfin-PDT, esophagogastroduodenoscopy (EGD) surveillance was performed at least every 2–3 weeks until the treatment-induced ulceration healed. Local efficacy was classified as L-CR or non-L-CR at each evaluation based on EGD findings. The criteria for L-CR were as follows: (1) confirmation of the disappearance of treatment-induced ulceration and scar formation, (2) no tumor endoscopically observed, and (3) disappearance of cancer cells histologically confirmed using a biopsy specimen when possible. After the patient had achieved L-CR, EGD was performed at least every 1–2 months until 6 months after Talaporfin-PDT. Chest/abdominal CT was performed at least every 3–6 months after Talaporfin-PDT. Lymph node and distant recurrence were defined as enlargement of a lymph node and a new lesion detected on CT, respectively.

### Statistical analysis

For the analysis of clinical characteristics and adverse events, Fisher’s exact test or the Wilcoxon’s rank sum test was used to identify differences between 2 groups. OS was defined as the time from Talaporfin-PDT to death from any cause, and it was censored at the date of the last follow-up for survivors. DSS was defined as the time from Talaporfin-PDT to death caused by esophageal cancer, and it was censored at the date of the last follow-up for survivors or death from any cause other than esophageal cancer. PFS was defined as the time from Talaporfin-PDT to the date of first recurrence or progression, or to the time of death from any cause, and it was censored at the date of the last visit for patients without progression. The survival curves were estimated using the Kaplan–Meier method, and P values were calculated using the log-rank test. The significance level of the P value was set at 0.05.

A logistic regression model was used to estimate the odds ratio and 95% confidence interval (CI) for non-L-CR. In selecting variables for the multivariate analysis, the results of univariate analyses were used only as a reference, and variables were primarily chosen based on their clinical importance and potential relevance to local control. In patients with multiple lesions, the main lesion was selected for analysis according to the following diagnostic criteria: (1) the lesion that did not achieve L-CR, (2) the deepest lesion identified in EUS or EGD findings, or (3) the largest lesion in EGD findings. All statistical analyses were performed using JMP® (SAS Institute Inc., Cary, NC, USA).

## Results

### Participants

**Fig. 2:**
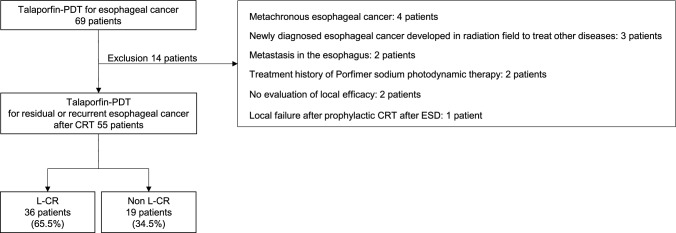
Flowchart of patient inclusion. Talaporfin-PDT: talaporfin sodium photodynamic therapy, *CRT*, chemoradiotherapy; *ESD*, endoscopic submucosal dissection; *L-CR*, local complete response

During the period under study, a total of 69 patients with esophageal cancer underwent Talaporfin-PDT in our hospital. We excluded 4 patients with metachronous esophageal cancer; 3 patients with newly diagnosed esophageal cancer developed in the radiation field used to treat pharyngeal cancer, lung cancer, or cervical lymph node tuberculosis; 2 patients with metastasis in the esophagus; 2 patients with a treatment history of porfimer sodium-PDT; 2 patients with no evaluation of local efficacy because of transfer immediately after treatment; and 1 patient with local failure after ER followed by prophylactic adjuvant CRT. The remaining 55 patients were included in this study. Among them, 36 (65.5%, 95% CI: 52.3–76.6) patients achieved L-CR (Fig. 2). It was confirmed histologically in 33 of the 36 patients that achieved LC-R.

Table 1 displays the demographic details of the 55 patients and the attributes of their lesions based on the local effects following talaporfin-PDT. Among the patients with ycT1 stage disease prior to talaporfin-PDT, 27 (75.0%) were in the L-CR group and 10 (52.6%) were in the non-L-CR group, with no significant difference observed (*P* = 0.132). The median laser dose of talaporfin-PDT was 450 J (range, 200–1200 J) for the L-CR group and 550 J (range, 300–700 J) for the non-L-CR group, with a significant difference noted (*P* = 0.045).

**Table 1:** Patients and lesion characteristics according to local effect after Talaporfin sodium photodynamic therapy (Talaporfin-PDT)

		Local effect after Talaporfin-PDT		
	Total	L-CR	Non L-CR	*P* value
	(*n* = 55)	(*n* = 36)	(*n* = 19)	
Gender (male/female)	(48/7)	(30/6)	(18/1)	0.401
Age (years old) (median, range)	74 (52–102)	73.5 (52–92)	74 (59–102)	0.936
ECOG performance status (0–1/> 2)	(55/0)	(36/0)	(19/0)	–
cT stage before initial treatment (1/2/3/4)	(29/11/8/7)	(21/6/4/5)	(8/5/4/2)	0.540
cN stage before initial treatment (0/1/2/3/X)	(30/10/11/2/2)	(25/4/6/0/1)	(5/6/5/2/1)	0.008
cM stage before initial treatment (0/1/X)	(49/5/1)	(33/3/0)	(16/2/1)	0.563
Histology (squamous cell carcinoma/adenocarcinoma)	(54/1)	(36/0)	(18/1)	0.346
Initial treatment (chemoradiotherapy/radiotherapy)	(45/10)	(32/4)	(13/6)	0.077
Tumor location (Ce-Ut/Mt/Lt)^a^	(9/27/19)	(7/17/12)	(2/10/7)	0.804
Endoscopic findings (superficial type/submucosal tumor type/ulceration type)^a^	(36/13/6)	(26/7/3)	(10/6/3)	0.345
ycT stage before Talaporfin-PDT (ycT1/ycT2)^a^	(37/18)	(27/9)	(10/9)	0.132
Maximum tumor diameter on endoscopic images (**≤ **15 mm/> 15 mm)^a^	(34/21)	(25/11)	(9/10)	0.148
Circumference of tumor (≤ 1/4/> 1/4)^a^	(36/19)	(26/10)	(10/9)	0.233
Esophageal stenosis before Talaporfin-PDT (absent/present)^a^	(47/8)	(33/3)	(14/5)	0.109
Laser dose (Joule) (median, range)^b^	500 (200–1200)	450 (200–1200)	550 (300–700)	0.045

^a^In patients with multiple lesions, the main lesion was selected

^b^In patients in which planed Talaporfin-PDT was performed in two phases for multiple lesions, the total dose of the two planed Talaporfin-PDT was listed

### Cumulative survival rates according to local effect

During the median follow-up period of 17.8 months (range, 1.7–69.3), 11 patients died of esophageal cancer, and 6 patients died of other causes. Local recurrence and lymph node/distant metastasis developed in 25 and 14 patients, respectively.

**Fig. 3:**
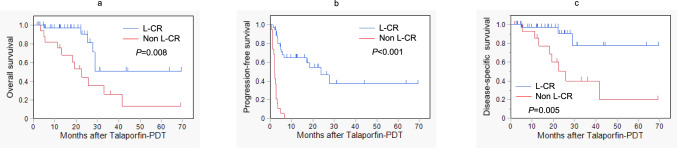
Cumulative survival rates according to local effect after talaporfin sodium photodynamic therapy (Talaporfin-PDT). **a** Overall survival. **b** Progression-free survival. **c** Disease-specific survival

The cumulative survival rates were analyzed in relation to the local effect (L-CR vs. non-L-CR), as depicted in Fig. 3. The OS rates at 1 and 3 years (Fig. 3a) were 97.0% and 50.9% for L-CR, compared to 75.6% and 26.5% for non-L-CR, respectively, showing a significant difference (*P* = 0.008). Similarly, the PFS rates (Fig. 3b) were 64.6% and 37.1% for L-CR, whereas non-L-CR was 0% at both time points, with a highly significant difference (*P* < 0.001). The DSS rates (Fig. 3c) were 97.0% and 77.6% for L-CR, compared to 85.7% and 40.0% for non-L-CR, respectively, also showing a significant difference (*P* = 0.005).

### Adverse events associated with Talaporfin-PDT according to local effect

Adverse events associated with Talaporfin-PDT were found in 81.8% of the study patients. The main adverse events were esophageal pain (58.2%), nausea (30.9%), fever (27.3%), esophageal stenosis (20.0%), hemorrhage (5.5%), and perforation (1.8%). There were no treatment-related deaths. Grade 3 hemorrhage according to the Common Terminology Criteria for Adverse Events (CTCAE) version 5.0 was observed in 1 patient with non-L-CR. Grade 4 perforation according to the CTCAE version 5.0 was observed in 1 patient with non-L-CR who developed life-threatening pneumonia caused by a tracheoesophageal fistula. The frequency of adverse events did not differ between the L-CR and non-L-CR groups (Table 2).

**Table 2:** Adverse events associated with Talaporfin sodium photodynamic therapy (Talaporfin-PDT) according to local effect

		Local effect after Talaporfin-PDT		*P* value
	Total	L-CR	Non L-CR	
	(*n* = 55)	(*n* = 36)	(*n* = 19)	
Esophageal pain	32 (58.2%)	19 (52.8%)	13 (68.4%)	0.389
Nausea	17 (30.9%)	10 (27.8%)	7 (36.8%)	0.548
Fever	15 (27.3%)	9 (25.0%)	6 (31.6%)	0.752
Stenosis	11 (20.0%)	5 (13.9%)	6 (31.6%)	0.161
Hemorrhage	3 (5.5%)	1 (2.8%)	2 (10.5%)	0.272
Perforation	1 (1.8%)	0 (0.0%)	1 (5.3%)	0.346
Skin photosensitivity	0 (0.0%)	0 (0.0%)	0 (0.0%)	–

Adverse events were graded according to the CTCAE version 5.0

### Treatment resistance factors associated with Talaporfin-PDT for local control

In univariate analyses, local resistance following Talaporfin-PDT showed a certain association with the initial treatment modality (treatment intensity), ycT stage prior to Talaporfin-PDT, and the presence of esophageal stenosis before the procedure. However, none of these factors achieved statistical significance (*P* = 0.071, 0.098, and 0.086, respectively). Although these variables were not statistically significant in the univariate analyses, they were considered clinically important factors that could potentially influence local control. Consequently, they were included in the multivariable model to minimize potential confounding. In multivariate analysis, esophageal stenosis before the procedure emerged as an independent predictor of local failure after Talaporfin-PDT, with an odds ratio of 5.626 (95% confidence interval: 1.051–30.124, *P* = 0.044) (Table 3). In the multivariate analysis of 54 patients, excluding those with adenocarcinoma, esophageal stenosis before the procedure was once again identified as an independent predictor of local failure after Talaporfin-PDT, with an odds ratio of 5.969 (95% confidence interval: 1.114–31.985, *P* = 0.037) (Supplementary Table S1).

**Table 3:** Treatment resistance factors associated with Talaporfin sodium photodynamic therapy (Talaporfin-PDT) for local control by logistic regression model

	Non L-CR (%)	Univariate		Multivariate	
		Odds ratio (95% CI)	*P* value	Odds ratio (95% CI)	*P* value
Gender					
Male	18/48 (37.5)	1 (ref.)			
Female	1/7 (14.3)	0.278 (0.031–2.497)	0.253		
Age					
≤ 75	10/30 (33.3)	1 (ref.)			
> 75	9/25 (36.0)	1.125 (0.369–3.431)	0.836		
cT stage before initial treatment					
T1	8/29 (27.6)	1 (ref.)			
≥ T2	11/26 (42.3)	1.925 (0.624–5.937)	0.254		
Initial treatment modality					
Chemoradiotherapy	13/45 (28.9)	1 (ref.)		1 (ref.)	
Radiotherapy	6/10 (60.0)	3.692 (0.893–15.274)	0.071	4.272 (0.939–19.427)	0.060
Tumor location					
Ce-Ut	2/9 (22.2)	1 (ref.)			
Mt	10/27 (37.0)	2.059 (0.356–11.906)	0.420		
Lt	7/19 (36.8)	2.042 (0.328–12.691)	0.444		
Macroscopic type					
Superficial type	10/36 (27.8)	1 (ref.)			
Submucosal tumor type	6/13 (46.2)	2.229 (0.600–8.275)	0.231		
Ulceration type	3/6 (50.0)	2.600 (0.448–15.092)	0.287		
ycT stage before Talaporfin-PDT					
ycT1	10/37 (27.0)	1 (ref.)		1 (ref.)	
ycT2	9/18 (50.0)	2.700 (0.834–8.741)	0.098	3.116 (0.865–11.224)	0.082
Maximum tumor diameter on endoscopic images					
≤ 15 mm	9/34 (26.5)	1 (ref.)			
> 15 mm	10/21 (47.6)	2.525 (0.803–7.945)	0.113		
Circumference of tumor					
≤ 1/4	10/36 (27.8)	1 (ref.)			
> 1/4	9/19 (47.4)	2.340 (0.734–7.456)	0.151		
Esophageal stenosis before Talaporfin-PDT					
Absent	14/47 (29.8)	1 (ref.)		1 (ref.)	
Present	5/8 (62.5)	3.929 (0.824–18.731)	0.086	5.626 (1.051–30.124)	0.044

**Fig. 4:**
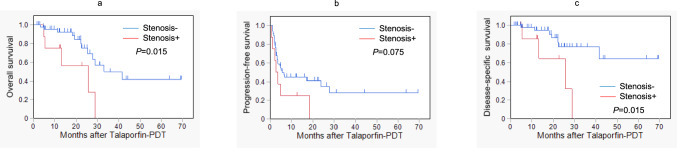
Cumulative survival rates in patients with or without esophageal stenosis before talaporfin sodium photodynamic therapy (Talaporfin-PDT). **a** Overall survival. **b** Progression-free survival. **c** Disease-specific survival

To identify any relationship between the factors associated with resistance and survival, we analyzed the cumulative survival rates of patients with or without esophageal stenosis prior to Talaporfin-PDT, as shown in Fig. 4. The OS rates at 1 and 3 years (Fig. 4a) were 75.0% and 0.0%, compared to 92.4% and 49.7%, respectively, with a significant difference noted at *P* = 0.015. The PFS rates (Fig. 4b) were 25.0% and 0.0% versus 44.6% and 28.0%, respectively, with no significant difference (*P* = 0.075). The DSS rates (Fig. 4c) were 85.7% and 0.0%, compared to 94.6% and 76.9%, respectively, with a significant difference found at *P* = 0.015.

## Discussion

This study investigated for the first time the treatment resistance factors associated with Talaporfin-PDT for local control after CRT for esophageal cancer. Esophageal stenosis before Talaporfin-PDT was an independent predictor of resistance to local control. Moreover, the 1-year OS and DSS rates were significantly lower in patients with stenosis than in those without stenosis. These results suggest that stenosis after CRT for esophageal cancer has a negative impact on the efficacy and prognosis for Talaporfin-PDT.

In a previous prospective study, the L-CR rate for Talaporfin-PDT was 88.5% [14]. A recent retrospective study reported an L-CR rate of 69% for Talaporfin-PDT given in the practice setting [15]. In the current study, the L-CR rate for Talaporfin-PDT was 65.5%. These data support the efficacy of Talaporfin-PDT for local control in the treatment of esophageal cancer after CRT. However, in this study, esophageal stenosis before Talaporfin-PDT affected the treatment efficacy, as shown by the poor L-CR rate of 37.5%.

In this study, eight patients with esophageal stenosis due to residual or recurrent esophageal cancer received Talaporfin-PDT, and L-CR was achieved in only three patients. Under the conditions without stenosis, an appropriate distance while maintaining a vertical view of the lesion by manipulating the angle of the endoscope is important for effective laser irradiation. However, under the condition with stenosis, it is difficult to maintain a vertical view of the lesion and an appropriate distance because the lumen is narrow and often deformed. These issues might be the reasons for the poor L-CR rate of the lesions with stenosis.

Even with a thin endoscope to irradiate lesions with stenosis, effective irradiation is often difficult. To solve this problem, it might be necessary to develop a new hood for the thin endoscope or a new irradiation probe. In addition, the difficulty of accurate evaluation of the invasion depth in the stenotic area using EUS and EGD might also affect the reason for this poor L-CR rate.

T stage before PDT not baseline (ycT) was reported to be associated with therapeutic effect of PDT [15, 16]. In this study, the L-CR rate tended to be higher in the ycT1 stage before Talaporfin-PDT than in ycT2 lesions (L-CR rate of ycT1: 73%, ycT2: 50%). In addition, L-CR rate of Talaporfin-PDT for the lesion with ycT1 and no stenosis was particularly good at 80.6%. This result indicated that Talaporfin-PDT might be good indication for such patients. Although the initial treatment modality did not reach statistical significance, the observed odds ratio (odds ratio 4.2, *P* = 0.060) suggests a potentially meaningful impact on local control; no statistically significant difference was detected, and the initial treatment may be of substantial importance. Notably, despite the fact that CRT had a greater number of including more cases with initially advanced cT categories before initial treatment than compared to RT group, the RT group demonstrated inferior poorer outcomes in the terms of local control of after PDT. Consequently, the disparity in the treatment intensity between CRT and RT may have affected the local control rate of PDT.

The frequencies of adverse events after Talaporfin-PDT, such as esophageal pain, fever, esophageal stenosis, skin photosensitivity, and perforation, have been reported as 53.8%, 30.8%, 4.5–7.7%, 0–4.5%, and 0%, respectively [14, 15]. In this study, the profile and frequency of adverse events were similar, and the frequency of adverse events did not differ between the L-CR and non-L-CR groups. However, grade 4 perforation was observed in 1 patient with non-L-CR who developed life-threatening pneumonia caused by a tracheoesophageal fistula. This patient had recurrent esophageal cancer within the esophageal stenosis caused by CRT. In this patient, it was difficult to maintain an adequate distance from the target lesion during the PDT procedure. In addition, the esophagus was irradiated circumferentially because of its narrow lumen. This situation may be associated with perforation after Talaporfin-PDT.

This study has several limitations. This was a single-center, retrospective study with a small number of patients, and the follow-up duration was short. In addition, this study included only one case of adenocarcinoma, which limited the ability to thoroughly evaluate the relationship between adenocarcinoma and treatment resistance factors. In the future, a nationwide real-world survey and a large-scale prospective study are needed to validate our results.

In conclusion, esophageal stenosis was an independent predictor of resistance to local control after Talaporfin-PDT and was associated with poor survival. The indications for the use of Talaporfin-PDT in patients with esophageal stenosis at the residual or recurrent esophageal cancer after CRT should be carefully considered. In addition, a new strategy for such cases is needed to improve the clinical outcomes.

## Supplementary Information

Below is the link to the electronic supplementary material.

Supplementary file1 (DOCX 18 kb)

## Data Availability

This study did not approved for external data sharing. Therefore, the data are not available beyond what is presented in the manuscript. Any future secondary use would require separate ethical committee’s approval.
