# Supplementary material for: Treatment resistance factors associated with Talaporfin sodium photodynamic therapy for local control after chemoradiotherapy for esophageal cancer
Source: Esophagus. 2026 Jan 16;23(2):391–9. doi: 10.1007/s10388-025-01177-w (PMC13038797; doi:10.1007/s10388-025-01177-w)
Supplement: Supplementary file 1 — Supplementary file1 (DOCX 18 kb) [file 10388_2025_1177_MOESM1_ESM.docx]

| **Supplementary Table 1. Treatment resistance factors excluding adenocarcinomas associated with Talaporfin sodium photodynamic therapy (Talaporfin-PDT) for local control by logistic regression model** | | | | | | |
| --- | --- | --- | --- | --- | --- | --- |
|  | | Non L-CR (%) | Univairate | | Multivariate | |
|  |  |  | Odds ratio (95%CI) | *P* value | Odds ratio (95%CI) | *P* value |
| Gender | Male | 17/47 (36.2) | 1 (ref.) |  |  |  |
|  | Female | 1/7 (14.3) | 0.294 (0.033-2.652) | 0.275 |  |  |
|  |  |  |  |  |  |  |
| Age | ≤75 | 10/30 (33.3) | 1 (ref.) |  |  |  |
|  | >75 | 8/24 (33.3) | 1 (0.320-3.123) | 1.000 |  |  |
|  |  |  |  |  |  |  |
| cT stage before initial treatment | T1 | 7/28 (25.0) | 1 (ref.) |  |  |  |
|  | ≥T2 | 11/26 (42.3) | 2.2 (0.692-6.992) | 0.181 |  |  |
|  |  |  |  |  |  |  |
| Initial treatment modality | Chemoradiotherapy | 12/44 (27.3) | 1 (ref.) |  | 1 (ref.) |  |
|  | Radiotherapy | 6/10 (60.0) | 4 (0.959-16.691) | 0.057 | 4.669 (1.022-21.332) | 0.047 |
|  |  |  |  |  |  |  |
| Tumor location | Ce-Ut | 2/9 (22.2) | 1 (ref.) |  |  |  |
|  | Mt | 10/27 (37.0) | 2.059 (0.356-11.906) | 0.420 |  |  |
|  | Lt | 6/18 (33.3) | 1.75 (0.275-11.152) | 0.554 |  |  |
|  |  |  |  |  |  |  |
| Macroscopic type | Superficial type | 9/35 (25.7) | 1 (ref.) |  |  |  |
|  | Submucosal tumor type | 6/13 (46.2) | 2.476 (0.656-9.344) | 0.181 |  |  |
|  | Ulceration type | 3/6 (50.0) | 2.889 (0.492-16.973) | 0.240 |  |  |
|  |  |  |  |  |  |  |
| ycT stage before Talaporfin-PDT | ycT1 | 10/37 (27.0) | 1 (ref.) |  | 1 (ref.) |  |
|  | ycT2 | 8/17 (47.1) | 2.4 (0.725-7.946) | 0.152 | 2.657 (0.716-9.860) | 0.144 |
|  |  |  |  |  |  |  |
| Maximum tumor diameter on endoscopic images | ≤15mm | 9/34 (26.5) | 1 (ref.) |  |  |  |
|  | >15mm | 9/20 (45.0) | 2.273 (0.709-7.284) | 0.167 |  |  |
|  |  |  |  |  |  |  |
| Circumference of tumor | ≤1/4 | 10/36 (27.8) | 1 (ref.) |  |  |  |
|  | >1/4 | 8/18 (44.4) | 2.08 (0.638-6.780) | 0.224 |  |  |
|  |  |  |  |  |  |  |
| Esophageal stenosis before Talaporfin-PDT | Absent | 13/46 (28.3) | 1 (ref.) |  | 1 (ref.) |  |
|  | Present | 5/8 (62.5) | 4.231 (0.881-20.309) | 0.072 | 5.969 (1.114-31.985) | 0.037 |
